# Energy Availability and Nutritional Intake during Different Training Phases of Wheelchair Athletes

**DOI:** 10.3390/nu15112578

**Published:** 2023-05-31

**Authors:** Anneke Hertig-Godeschalk, Belinda Ruettimann, Ezra Valido, Marija Glisic, Jivko Stoyanov, Joelle L. Flueck

**Affiliations:** 1 Swiss Paraplegic Research, 6207 Nottwil, Switzerland; 2 Institute of Sports Medicine, Swiss Paraplegic Centre Nottwil, 6207 Nottwil, Switzerland; 3 Department of Health Sciences, University of Lucerne, 6002 Lucerne, Switzerland; 4 Institute of Social and Preventive Medicine, University of Bern, 3012 Bern, Switzerland

**Keywords:** paraplegic, tetraplegic, nutrition, Paralympic, season, spinal cord injury, macronutrient, micronutrient

## Abstract

Optimizing nutritional intake and timing helps athletes to improve performance and long-term health. Different training phases can require varying nutritional needs. In this study, we conducted a descriptive assessment of dietary intake, energy availability (EA), and blood biochemical parameters in elite wheelchair athletes during distinct training phases. Data analyzed in this study were collected as part of a randomized controlled crossover trial exploring the feasibility of probiotics and prebiotic supplementation. Data were obtained from consecutive three-day diaries and blood samples, both collected at four different time points across four consecutive months. We included 14 athletes (mean (standard deviation) age 34 (9) years, eight females, and six males) active in different wheelchair sports. The mean daily nutritional intake (g/kg body mass) for females and males was 2.7 (0.9) and 4.0 (0.7) for carbohydrates, 1.1 (0.3) and 1.5 (0.3) for protein, and 0.8 (0.3) and 1.4 (0.2) for fat. EA did not change across the four time points in either female (*p* = 0.30) or male (*p* = 0.05) athletes. The mean EA was lower in female athletes compared to male athletes (*p* = 0.03). Low EA (≤30 kcal/ kg fat-free mass/day) was observed in female (58 (29) % of days) and male (34 (23) % of days) athletes. Iron deficiency with anemia was observed in two female athletes. Mean vitamin D levels were insufficient (<75 nmol/L). Macronutrient intake, EA, and blood biochemical parameters were suboptimal in this cohort of elite wheelchair athletes, especially in female athletes.

## 1. Introduction

To maintain a long-term and successful career, elite athletes try to prevent health problems and maximize training adaptations. This includes not only managing training volume and intensity, along with recovery, but also tailoring nutrition to individual needs [1]. Nutritional intake depends on factors such as training content, intensity, duration, and overall load, as well as individual goals such as optimizing body composition for peak performance in a major competition. An important indicator of the balance between training and nutrition is energy availability (EA). EA refers to the energy that remains to support optimal health and body function after subtracting exercise energy expenditure (EEE) from energy intake (EI) [2]. An EA of 30 kcal/kg fat-free mass (FFM)/day or less is defined as low energy availability (LEA) in able-bodied athletes [3]. Prolonged LEA can impair training, recovery, and performance and may result in health issues such as low bone mineral density, micronutrient deficiencies, and menstrual and hormonal dysfunction [1,3,4,5].

While research on EA in able-bodied athletes is emerging, studies in wheelchair athletes are scarce [6,7,8,9]. Depending on the impairment, wheelchair athletes may exhibit differences in musculoskeletal and gastrointestinal function, as well as body composition, compared to able-bodied athletes [10,11,12,13,14]. These differences may also affect resting energy expenditure (REE), EEE, and ultimately EA. Lower REE (up to 27% less) and EEE (up to 75% less) have been observed in individuals with spinal cord injuries (SCI) compared to able-bodied individuals, although most of these findings are from studies in non-athletic populations and show conflicting results for REE [7,10,13,15]. The extent of these differences depends on the lesion level and completeness of the SCI. Due to these differences, guidelines regarding EI, intake timing, and thresholds for LEA originally designed for able-bodied athletes may not be directly applicable to wheelchair athletes [7,10,13,14,16]. Particularly for athletes with SCIs, protein and carbohydrate requirements may vary depending on their lesion characteristics and comorbidities [17,18]. Nevertheless, dietary guidelines for wheelchair athletes are generally based on recommendations for able-bodied athletes [7,11,12,17,18]. LEA has been associated with impaired iron metabolism, including iron deficiency [19]. Assessing micronutrient levels and immune status, in addition to EA, provides a better understanding of an athlete’s overall health. Furthermore, correction of ferritin and vitamin D deficiencies may improve athletic performance [1,19,20,21,22,23].

Although cut-off values for LEA in wheelchair athletes have yet to be established, it appears to be prevalent in this population [8,9]. Studies assessing EA at different time points are lacking. Training volume and intensity, as well as body composition goals, may change during different training phases [24]. Therefore, EA may also vary. This makes the assessment of EA across different training phases of the athletic season particularly relevant. Our study aimed to assess dietary intake, EA, and blood biochemical parameters at four consecutive time points during the pre-competition and competition phases in elite wheelchair athletes participating in a pilot feasibility study.

## 2. Materials and Methods

### 2.1. Setting and Study Population

Data analyzed in this study were collected between March and October 2021 as part of a pilot study for which the protocol, participant flowchart, and feasibility results have been published elsewhere [25,26]. This pilot study aimed to assess the feasibility of a randomized controlled crossover trial investigating the effects of probiotic and prebiotic supplementation on the health of elite Swiss wheelchair athletes. Athletes received either daily probiotic or prebiotic supplementation for four weeks, followed by a four-week washout period, and another four weeks of daily supplementation with the other supplement. Written informed consent was obtained from all participants. The study was conducted in accordance with the Declaration of Helsinki, approved by the Swiss Ethics Committee for Northwest/Central Switzerland (EKNZ, project ID: 2020-02337) and registered at ClinicalTrials.gov (NCT04659408). As part of the standard support for elite wheelchair athletes in our department, all athletes have received recommendations from a qualified nutritionist at least once a year. This included advice on how to optimize nutritional intake around training and competition.

### 2.2. Data Collection

Prior to the first time point, a full-body dual-energy X-ray absorptiometry (DXA) was performed using a Lunar iDXA scanner (GE Healthcare Lunar, Madison, WI, USA) to assess body composition. Baseline characteristics including height, type of sport, and—if applicable—lesion characteristics were collected at the first time point. Further measurements took place at each of the four time points; T0 = baseline, T1 = 4 weeks, T2 = 8 weeks, and T3 = 12 weeks. Athletes were instructed to record their weighed food intake and photograph their meals for three consecutive days prior to each of the time points. Similarly, exercise information was recorded for three consecutive days prior to each time point on a paper form or in an online training diary program. Details on time, duration, type of sport, and intensity were documented. Training intensity was rated using one out of four intensities according to Hottenrott [27]: recovery, basic endurance, moderate endurance, or submaximal to maximal work (interval training). All dietary and training diaries were reviewed by qualified study personnel at each time point, and entries were discussed and corrected with the athlete as needed. As the measurement time points took place at different days of the week, dairies included entries from weekdays and weekends, as well as training days and non-training days. At each time point, body mass (BM) was assessed by measuring the athlete separately from their wheelchair. A fasting blood sample was obtained from the antecubital vein at each time point. The following parameters were measured: hemoglobin (photometric method, XN-1000, Sysmex, Switzerland), ferritin (latex particle-enhanced turbidimetric immunoassay (LETIA), Cobas 6000 c501, Roche, Switzerland), c-reactive protein (CRP, latex particle-enhanced turbidimetric immunoassay (LETIA), Cobas 6000 c501, Roche, Switzerland), and vitamin D (25(OH)D, electrochemiluminescence immunoassay (ECLIA), Cobas 6000 e601, Roche, Switzerland). At each time point, the frequency of 36 GI symptoms during the previous two weeks was measured on a four-point Likert scale (ranging from 0 = “all the time” to 4 = “never”) using the Gastrointestinal Quality of Life Index (GIQLI) questionnaire [28].

### 2.3. Data Preparation

EEE was calculated based on the energy expenditure recommendations of Conger and Basset [29]. Nutrient analysis software (PRODI 6.11, Nutri-Science GmbH, Stuttgart, Germany) was used to calculate EI and macronutrient intakes. Relative nutritional intakes (g/kg BM) were calculated for carbohydrates, protein, and fat. Compliance with dietary recommendations was assessed for the intake of carbohydrates (3–12 g/kg BM), protein (>1.2 g/kg BM), and fat (20–35% of total intake) [1,17]. The timing of carbohydrate intake in the last 1–4 h before exercise (1–4 g/kg BM) and protein intake within one hour after exercise (20–30 g) was analyzed. EA was calculated as EI (kcal/day) minus EEE (kcal/day) relative to the FFM [30]. EA was categorized as “LEA” (≤30 kcal/kg FFM/day) [3]. EEE, EI, and EA were calculated over the three consecutive days at each time point as well as over the entire study. Anemia was defined as a hemoglobin level <120 g/dL (females) or <140 g/dL (males) [21]. Iron deficiency was defined as a ferritin level <30 μg/L [21]. Vitamin D levels below 75 nmol/L were defined as insufficient [31].

### 2.4. Data Analyses

Mean and standard deviation (SD) were calculated for all parameters. Shapiro–Wilk tests and Mauchly’s Tests of Sphericity were run to test for the violation of assumptions of normality and sphericity (*p* > 0.05). Assumptions were violated for BM and carbohydrate intake in both sexes, and for EEE in females. Accordingly, differences between sexes, lesion levels, and sports were assessed using t-tests or Wilcoxon–Mann–Whitney tests. Differences in parameters across time points were assessed with one-way repeated measures ANOVA, one-way repeated measures ANOVA with Greenhouse–Geisser correction, or Friedman’s tests. Correlations were assessed by Pearson’s correlation. A *p*-value below 0.05 (two-tailed) was considered statistically significant. Analyses were performed using Stata (StataCorp. 2017, Stata Statistical Software: Release 16.1. StataCorp LLC: College Station, TX, USA).

## 3. Results

### 3.1. Athlete Characteristics

Fourteen athletes (mean (SD) age 34 (9) years) participated in this study (Table 1) and there were no dropouts. Most athletes were female (n = 8) and had a traumatic SCI (n = 6). BM was stable at all four time points for both female and male athletes (*p* ≥ 0.51). No athlete was on a weight loss diet and only one athlete was on a vegetarian diet. GIQLI scores throughout the study indicated a low frequency of GI complaints [26]. All athletes competed at an international level and eight participated in the Paralympics. Athletes participated in a variety of sports, with the majority participating in outdoor sports (n = 11) and handcycling (n = 4). As most athletes competed in summer sports, the study took place during the pre-competition and competition seasons for most of the athletes. Additional athlete characteristics have been published previously [26].

### 3.2. Energy Expenditure and Intake

**Figure 1:**
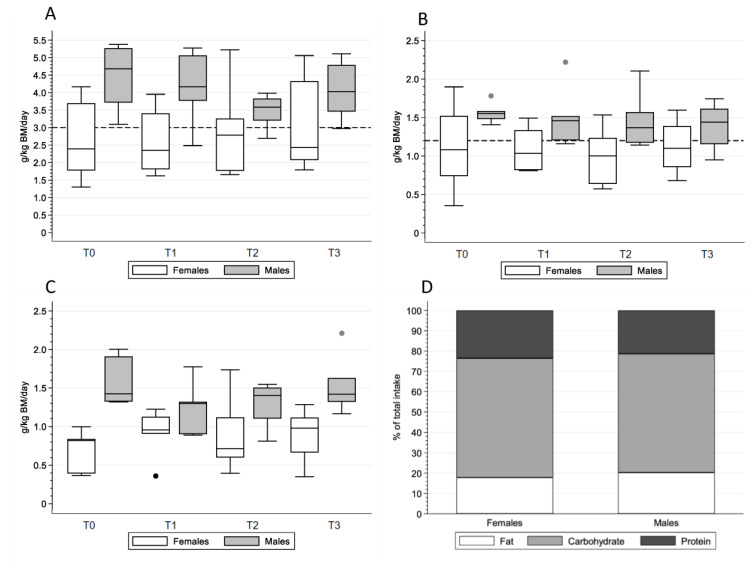
Macronutrient intake adjusted by body mass (BM) at each time point for (**A**) carbohydrate, (**B**) protein, and (**C**) fat. The dashed lines in A and B represent the minimum recommended intake. (**D**) displays the distribution of macronutrient intake over all time points. T0 = baseline, T1 = 4 weeks, T2 = 8 weeks, and T3 = 12 weeks.

EEE and EI were similar across the four time points, and also within both sexes (*p* ≥ 0.10). Relative carbohydrate intake was similar across time points in male athletes, with values decreasing from time point T0 (4.5 (0.9) g/kg BM) to T2 (3.5 (0.5) g/kg BM) and increasing again at the last time point (4.1 (08) g/kg BM, *p* = 0.05, Figure 1A). Across the four time points, relative carbohydrate intake did not differ in female athletes (*p* = 0.75). Relative protein intake was similar across the time points, and also within both sexes (*p* ≥ 0.42, Figure 1B). The relative fat intake was similar across time points in both male and female athletes (*p* ≥ 0.19, Figure 1C).

When the three-day diaries from all time points were combined, the mean EEE was 344 (139) kcal/day. Mean EI was 1674 (481) kcal/day (Table 2). The overall relative percentage fat intake was 18 (28)% in females and 21 (2)% in males (Figure 1D). When comparing athletes with tetraplegia and paraplegia, mean EEE, EI, and macronutrient intakes by BM were similar (*p* ≥ 0.17).

**Figure 2:**
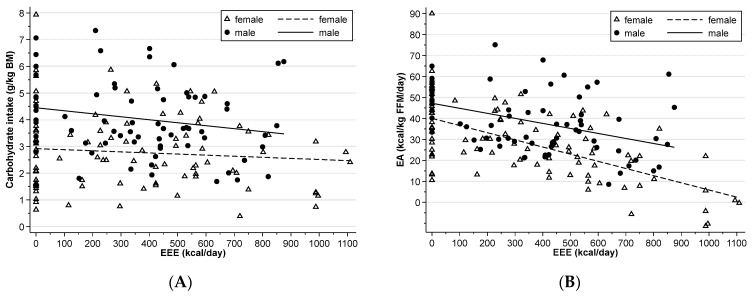
Exercise energy expenditure (EEE) by (**A**) carbohydrate intake by body mass (BM) and (**B**) energy availability. Data from each of the three days for each of the four time points are shown.

Relative carbohydrate intake did not correlate with EEE (*p* = 0.13, Figure 2A). On rest days, carbohydrate was higher than on days with higher EEE in some athletes. Daily carbohydrate intake recommendations were met more often by male than female athletes (*p* = 0.02, Table 2). Daily protein intake recommendations were met more often by male compared to female athletes (*p* = 0.01). Daily fat recommendations were not met in both female and male athletes equally (*p* = 0.67), with most athletes having intakes below recommendations. A carbohydrate intake of 1–4 g/kg BM within 1–4 h before endurance training was achieved in 53 (32)% of training sessions, with similar numbers in female and male athletes (54 (29) vs. 51 (36)%, *p* = 0.86). Protein intake of 20–30 g within 1 h after intensive or strength training was achieved in 34 (29)% of sessions, with lower numbers in female compared to male athletes (14 (20) vs. 58 (17)%, *p* = 0.004).

### 3.3. Energy Availability

**Figure 3:**
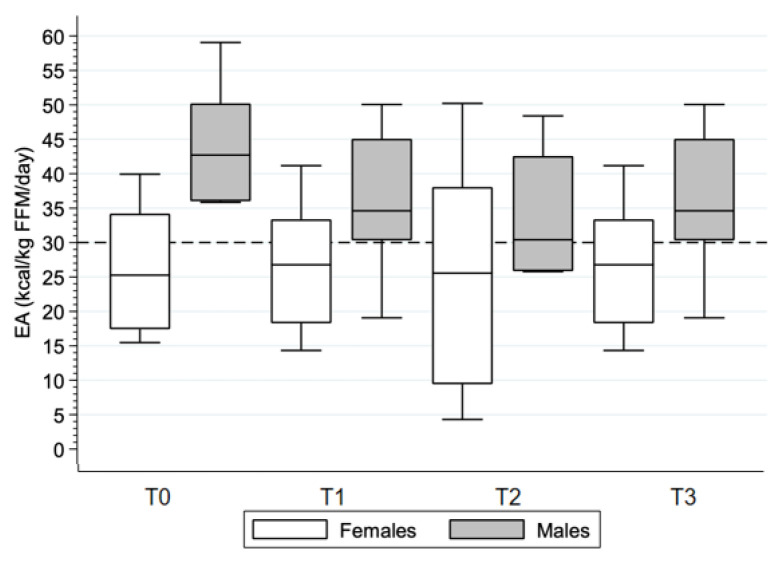
Energy availability across time points. The cutoff for low energy availability is indicated at 30 kcal/kg fat-free mass/day. T0 = baseline, T1 = 4 weeks, T2 = 8 weeks, and T3 = 12 weeks.

Although not significant (*p* = 0.05), EA fluctuated across time points in male athletes, with values decreasing from time point T0 (44 (10) kcal/kg FFM/day) to T2 (34 (9) kcal/kg FFM/day) and increasing again at the last time point (41 (10) kcal/kg FFM/day, Figure 3, Table 3). EA across time points was similar in female athletes (*p* = 0.30).

The mean EA across all time points was 32.1 (10.2) kcal/ kg FFM/day with lower values in female compared to male athletes (*p* = 0.03, Table 3). Mean EA was similar in athletes with tetraplegia and those with paraplegia (*p* = 0.40). LEA was prevalent for at least one day in every athlete. The percentage of LEA days was similar between female and male athletes (*p* = 0.12). Athletes with tetraplegia had a similar percentage of LEA days compared to athletes with paraplegia (46 (22)% vs. 36 (28)% of days, *p* = 0.54). Handcycling athletes had a similar percentage of LEA days compared to athletes in other sport disciplines (25 (6)% vs. 56 (29)% of days, *p* = 0.06).

Large variations in daily EA were found in most athletes, especially in female athletes (Table 3). The largest range was found in athlete F08, whose EA ranged from −11 to 90 kcal/kg FFM/day. A moderate negative correlation was found between daily EA and EEE (r = −0.53, n = 159, *p* < 0.001, Figure 2B). This correlation was moderate in female (r = −0.63, n = 88, *p* < 0.001) and small in male (r = −0.42, n = 69, *p* < 0.001) athletes.

### 3.4. Blood Biochemical Parameters

**Figure 4:**
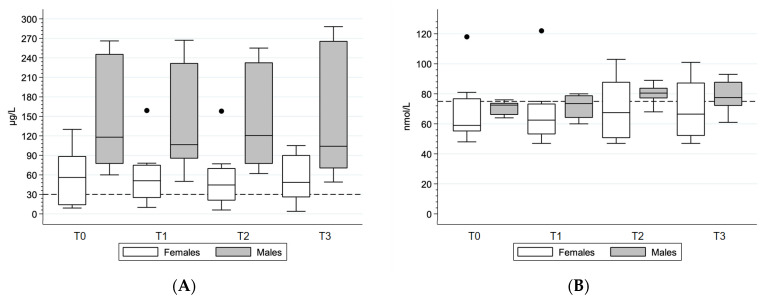
Blood biochemical parameters across time points for (**A**) ferritin (cutoff deficient levels indicated at 30 μg/L) and (**B**) vitamin D (cutoff insufficient levels indicated at 75 nmol/L) levels.

CRP levels exceeded 5 mg/l in five athletes in three time points, with a maximum value of 12 mg/L. Mean ferritin levels were 56 (43) μg/L in female and 145 (90) μg/L in male athletes (Figure 4A). Insufficient ferritin levels were observed in three female athletes but none of the male athletes (Table 4). Two female athletes had an iron deficiency with anemia, with one of them demonstrating this at all time points. Mean vitamin D levels were insufficient (72 (17) nmol/L). Only one athlete maintained sufficient vitamin D levels at all time points. Vitamin D levels increased slightly during the summer months (generally T2 and T3) in male athletes but less in female athletes (Table 4, Figure 4B).

## 4. Discussion

We evaluated EI and EA at four distinct time points during the athletic season in wheelchair athletes. Most athletes competed in summer sports. As data collection took place from spring until fall, this represents pre-competition and competition seasons for most of the athletes. Neither EA nor EI displayed significant differences across the various time points. Interestingly, all of the athletes experienced LEA for at least one day, indicating how tough fueling is for elite athletes. Furthermore, daily macronutrient intake and timing were frequently suboptimal, with athletes not adjusting EI to accommodate higher training loads. The results are somewhat surprising considering that these are all professional athletes receiving professional support, and it therefore might be assumed that they have the nutritional knowledge and support to tailor their nutrition to their needs. However, our findings align with the reality of professional sport and demonstrate significant potential for improvement in sports nutrition support and implementation.

### 4.1. Energy Availability and Intake across Athletic Seasons

Optimizing body composition should ideally be achieved before the start of the competitive season [1]. In the case of a planned BM loss, this allows fat mass to be lost over a longer period with minimal energy deficit or impact on performance, while avoiding a yo-yo effect or weight regain. In our study, none of the athletes were on a specific weight loss diet and BM remained stable at all time points. Unfortunately, body composition was only assessed once at the beginning of the study. It is possible that body composition changed during the study to achieve peak performance during the season, without a change in BM. In a nine-year case study of a female middle-distance runner, an optimized periodization approach for body composition and EA correlated with long-term health and steady performance improvement [32].

The EI we observed, even the notably low values in some of our female athletes, is consistent with findings from previous studies in wheelchair and Paralympic athletes [8,9,33,34,35]. This consistency reinforces our hypothesis that the low EI may be a contributing factor to the observed high prevalence of LEA in our athletic cohort. Nevertheless, energy requirements vary between days and across phases of the athletic season, with higher dietary intakes required during periods of high training loads [1,30,36]. Especially during the competition phase, EA should be optimized to maintain health and performance while reducing the risk of injury. Consistent with previous studies in wheelchair athletes [8,9], we found large day-to-day fluctuations in EA. However, EEE was similar across time points. Furthermore, EA remained similar across all four time points in the male athletes. In female athletes, EA was low throughout the study and indicative of LEA at three time points (Table 3). The main reason for the LEA was an overall low EI (1343 ± 257 kcal/d) in these athletes. In contrast, a study of 88 (24 female) elite able-bodied athletes competing in different sports found higher EA during the competition phase than in the pre-competition phase [37]. In these athletes, LEA was found in 13% (2/88 females) of the athletes in the pre-competition phase but in none during the competitive phase. Especially during a competitive phase, an optimal EA is crucial to perform and recover well. In our study, athletes did not adjust carbohydrate intake to training load, leading to an even lower EA with higher training volume or intensity. In some athletes, carbohydrate intake was indeed higher on rest days than on training days. This shows, again, how much potential these athletes have regarding optimal health, performance, and recovery. In comparison, professional able-bodied male cyclists seem to adjust their EI much better to EEE [38]. However, the increased EI was still insufficient to meet the higher energy demands, resulting in suboptimal EA. Again, this highlights how difficult fueling can be for professional athletes and how well-planned and structured nutrition must be [4]. A review of able-bodied endurance athletes indicated that female athletes are even worse at adapting EI to EEE, especially during the competitive phase [24]. This puts female athletes at particularly high risk for LEA. Even a few days of LEA can affect performance, while prolonged LEA can pose serious health risks [1,4]. Female athletes with SCI are no exception to this and may be at an even higher risk for LEA [6].

### 4.2. Macronutrient Intake across Athletic Seasons

Adequate daily protein intake (>1.2 g/kg BM) is essential for athletes to induce training adaptations and facilitate recovery during all training phases, especially to compensate for the high muscle protein turnover rate [1,36]. Our results showed that male athletes were aware of this, as protein intake was adequate (1.5 (0.3) g/kg BM) at all four time points. In female athletes, however, protein intake was at the lower end of the recommendations (1.1 (0.3) g/kg BM). Another study in elite athletes with SCI (19/39 females) found a similar protein intake during the pre-competition (1.2 g/kg BM) and the post-competition phase (1.4 g/kg BM) [34].

Unlike protein, carbohydrate intake must be periodized according to training load or individual goals [1]. It might be lower if an athlete is trying to lose weight. However, during the competition phase, athletes should not reduce carbohydrate intake to prevent underfueling. As carbohydrates are used as a primary energy source during high-intensity work, one would not want to put their performance at risk [36]. Carbohydrate intake across time points showed some variation in our male athletes (range 3.5 to 4.5 g/kg BM), but less so in our female athletes (range 2.6 to 3.1 g/kg BM). Furthermore, carbohydrate intake was relatively low, considering that 3 g/kg BM per day is considered the low end of the recommended carbohydrate intake for athletes, for example on training days with very low volume or intensity [1]. However, our athletes also consumed insufficient amounts of carbohydrates on training days of moderate to high training volume or intensity. Similar findings were reported in another study performed with athletes with SCI [34]. Here, carbohydrate intake was reported to be similar in both the post-competition (3.5 (1.2) g/kg) and the pre-competition (3.1 (0.8) g/kg) phases. The latter study did not assess EEE so the carbohydrate could not be placed in a broader perspective. Nevertheless, it appears that there is a need to optimize carbohydrate intake according to the athletes’ needs. Female athletes especially tend to fear carbohydrate intake due to concerns about weight gain, which puts them at an increased risk of LEA [39].

### 4.3. Blood Biochemical Parameters

Iron deficiency without anemia impairs oxidative capacity, leading to health and exercise complications [1,19,40]. In iron deficiency with anemia, iron deficiency is accompanied by compromised hemoglobin levels, which limits oxygen transport to the muscle and further impairs exercise performance [19]. Iron deficiency is more prevalent in athletes compared to non-athletes due to exercise-induced effects on iron metabolism, inadequate iron intake, and generally low EI [19]. Long-term effects of LEA, including menstrual and hormonal dysfunction, may further impact iron stores [19,40]. Data on the prevalence of iron deficiency in wheelchair athletes are scarce. A study of 13 female wheelchair basketball players reported ferritin levels of 32 (29) μg/L [41], which is lower than the levels we found in our female athletes (56 (43) μg/L). In our study, three female athletes but none of the male athletes showed iron deficiency without anemia. Two other female athletes presented with iron deficiency with anemia. One of them showed these symptoms at all four time points but did not want to receive oral iron supplementation. Our findings may reflect results in able-bodied athletes, as iron deficiency is also more common in females (up to 47%) compared to males (up to 17%) in this population [19,40]. Better strategies and more education are needed to prevent athletes from developing iron deficiencies, especially those at a higher risk.

Vitamin D plays a role in musculoskeletal and immune function. Therefore, maintaining sufficient levels is essential for para-athletes and able-bodied athletes [42]. Due to reduced physical activity and sun exposure, as well as physiological adaptations and medication use, insufficient vitamin D levels are more prevalent in individuals and athletes with SCI compared to their able-bodied counterparts [43,44]. It is therefore not surprising that we found that all but one athlete had insufficient vitamin D levels at all time points. This is in line with another study showing insufficient vitamin D levels in 39 athletes with SCI in autumn and in winter months (69.6 (19.7) vs. 67.4 (25.5) nmol/L) [45]. In 20 indoor athletes with SCI, mean vitamin D levels during winter months were even lower (44 (18) nmol/L) [46].

Several studies in athletes with SCI and other para-athletes have reported insufficient iron and vitamin D intakes [34,35,47]. Reduced total EI, including vitamin D-rich foods such as fish or fortified milk and iron-rich foods such as meat, may increase the risk of low micronutrient levels. Insufficient iron or vitamin D levels should be prevented to maintain overall health and performance [1,11,42]. Regular screening of these biochemical parameters may help to prevent insufficiencies.

### 4.4. Strengths and Limitations

This is the first study evaluating EA during different training phases of wheelchair athletes. One limitation of our study is that not all athletes competed in the same sport type, resulting in unmatched time points in terms of the athletic season. However, this bias was minimized as most athletes competed in summer sports. Therefore, most athletes were in the pre-competition phase at the first two time points and in the competition phase at the last two time points. Nutrition knowledge was not assessed in this study. It may be that some of our athletes, such as endurance athletes, are more aware of the importance of fueling, especially carbohydrate intake [48]. Since all of our male athletes were active in endurance sports, this may explain some of the variation. A comparison between different sport types, for example between athletes participating in summer and winter sports, would be interesting for future studies.

DXA measurements have been identified as the most accurate tool to assess whole-body fat, regional fat mass, and FFM in adults and athletes with SCI [49,50]. As we did not find any change in BM over the four time points, it would have been interesting to see if there were any changes in body composition during this period. Nonetheless, including more DXA measurements was not feasible due to the increased burden and cost, including radiation exposure.

Data collection using diaries is subject to under- or over-reporting, which may have influenced our results. The collection of physical activity data based on self-reported training diaries may lead to over- or underestimation of EEE and thus EA [51]. As most of the athletes are regularly supervised during training and undergo exercise testing in our department, we know that the data collected in our study correspond to the actual training. All diaries were reviewed by qualified study personnel and photographs were provided together with the diary to reduce bias. Similar results regarding EI in female wheelchair athletes were found in another study within our department [9]. These and our new data clearly show that female athletes are restricting carbohydrate intake and not energy intake per se. Furthermore, despite the low EA, BM remained similar over the course of the study. This may indicate that the EA, and therefore the EI, we found is indeed correct and reflects a longer-term problem that already existed before the study began [52]. For these reasons, we speculate that underreporting was not widespread among our athletes. However, we are aware that underreporting in food diaries is a common bias in most studies [7,53]. We posit that a three-day data collection period, including both weekdays and weekends as well as training and non-training days, can mitigate potential bias and provide a reliable representation for the analysis of a high-quality weighed food record [54]. Nevertheless, we acknowledge that data collected over an entire week may be even more comprehensive in an athletic population given the daily variation in training regimens. Moreover, without any data on the risk of eating disorders, we cannot exclude eating disorders as an explanation for low EI. Micronutrient intake was not assessed in this study, preventing us from evaluating potential associations with ferritin or vitamin D intake, and blood status or supplementation recommendations.

It remains unknown whether the cut-off values for LEA in able-bodied athletes can be applied to wheelchair athletes [14]. This complicates the classification of EA in our athletes. Nevertheless, comparisons between sexes or different time points were not affected by this, as we applied the same analysis and cut-offs to all measurements.

The data analyzed in this study were collected as part of a clinical trial. The athletes were asked not to change their diet or exercise patterns. The intervention evaluated in the clinical trial, the intake of probiotic and prebiotic supplementation, was also not expected to influence diet and exercise patterns. Nevertheless, participation in a clinical trial may have affected the behavior of the athletes. We speculate that this only had a minimal impact on the findings reported in this study.

### 4.5. The Future of Nutrition in Wheelchair Athletes

Dietary guidelines for able-bodied individuals may not be directly transferable to wheelchair athletes due to differences in energy requirements, intestinal dysmotility, and other physiological adaptations [7,10,13,14,16]. In the absence of specific guidelines for wheelchair athletes, we compared the nutritional intake of our athletes with the guidelines for able-bodied athletes. Nevertheless, the sports nutrition practices revealed in this study highlight significant potential for improvement. Providing information and education regarding sports nutritional knowledge to these athletes may be a possible solution. A survey in an international cohort of athletes with SCI found a modest knowledge of sports nutrition (<60% of questions answered correctly), on topics such as weight management, timing, quality, and quantity of macronutrient intake [48]. The internet was ranked as the primary source of information, with advice from nutritional experts ranked second. This is yet another indication of where sports nutrition professionals need to focus their efforts in the future.

A study involving six athletes with lower-limb disabilities reported that the athletes did not receive regular advice from a dietitian [55]. Although nutritional knowledge was not assessed in our study, all athletes received recommendations from a sports nutritionist at least once a year. Nutritional guidance after the onset of SCI tends to focus on weight management, as energy requirements are lower than before SCI onset [7]. As patients become athletes, they need specific guidance on how to adapt and optimize their nutrition to align with their training schedules. Athletes may still be concerned about weight gain and may tend to restrict calories even as they increase the frequency and intensity of their exercise. This was also observed in a survey of 260 para-athletes, where 47% considered themselves overweight and 44% felt pressure to maintain BM [56]. Moreover, 62% of the athletes in the latter survey study were currently trying to change their body composition or lose weight to enhance their performance. This underscores the importance of monitoring any intentional changes to body composition or BM by a qualified sports nutritionist [52].

Our study highlights the need for specific nutritional guidelines tailored to wheelchair athletes, as well as the importance of continuous education and guidance from qualified sports nutritionists. Further research is necessary to develop and implement these guidelines to support the unique needs and demands of this population.

## Figures and Tables

**Table 1:** Athlete characteristics.

	Overall	Females (n = 8)	Males (n = 6)
Age (years)	34 (9)	32 (11)	36 (8)
Height (cm)	165 (13)	159 (14)	172 (7)
Body mass (kg)	58 (10)	59 (12)	58 (7)
BMI (kg/m²)	22 (4)	23 (5)	20 (2)
Fat mass (kg)	18 (8)	22 (8)	13 (4)
FFM (kg)	40 (8)	36 (5)	46 (7)
Diagnosis (n(%))			
Traumatic SCI	6 (43)	2 (25)	4 (67)
Meningomyelocele	5 (36)	3 (38)	2 (33)
Multiple sclerosis	2 (14)	2 (25)	0 (0)
Arthrogryposis	1 (7)	1 (13)	0 (0)
Time since injury (years)	19 (4)	18 (6)	20 (4)
NLI (n(%))			
Tetraplegia	4 (36)	2 (40)	2 (33)
Paraplegia	7 (64)	3 (60)	4 (67)
AIS (n(%))			
A	5 (56)	2 (50)	3 (60)
B–C	3 (33)	1 (25)	2 (40)
D	1 (11)	1 (25)	0
Mean training duration (hours/week)	14 (5)	14 (5)	14 (6)

AIS = American spinal injury association impairment scale, BMI = body mass index, FFM = fat-free mass, NLI = neurological level of injury, and SCI = spinal cord injury. Data are reported as mean (standard deviations) unless indicated otherwise.

**Table 2:** Mean nutritional intake across all four time points.

	All (n = 14)	Females (n = 8)	Males (n = 6)
Daily energy intake			
Total energy intake (kcal)	1674 (481)	1343 (257)	2116 (315)
Daily Carbohydrate Intake			
Total intake (g)	190 (56)	156 (41)	235 (38)
Relative intake (g/kg BM)	3.3 (1.0)	2.7 (0.9)	4.0 (0.7)
Recommended intake 3–12 g/kg BM (% of days)	57 (36)	39 (34)	80 (22)
Daily Protein Intake			
Total intake (g)	73 (19)	62 (13)	87 (17)
Relative intake (g/kg BM)	1.3 (0.3)	1.1 (0.3)	1.5 (0.3)
Recommended intake >1.2 g/kg BM (% of days)	52 (30)	36 (24)	74 (24)
Daily Fat Intake			
Total intake (g)	63 (23)	47 (14)	83 (15)
Relative intake (g/kg BM)	1.1 (0.4)	0.8 (0.3)	1.4 (0.2)
Recommended intake 20–35% of total energy (% of days)	42 (23)	40 (28)	45 (15)

BM = body mass. Data are reported as mean (standard deviations).

**Table 3:** Energy availability.

	Sport	Mean (SD) EA over Three Days				Mean (SD) EA All Time Points	Number of Days LEA
		T0	T1	T2	T3		
F01	Cycling	29.6 (11.8)	26.8 (7.7)	50.2 (7.6)	43.3 (7.0)	37.5 (11.1)	3/12
F02	Tennis	24.7 (2.1)	30.1 (9.7)	38.1 (5.5)	30.8 (12.6)	30.9 (5.5)	6/12
F03	Badminton	15.5 (5.7)	.	9.4 (4.4)	10.2 (10.7)	11.7 (3.3)	9/9
F04	Athletics	17.7 (4.7)	18.3 (9.4)	.	24.9 (18.7)	20.3 (4.0)	8/9
F05	Shooting	17.2 (6.8)	41.2 (15.2)	25.6 (5.7)	42.0 (17.6)	31.5 (12.2)	7/12
F06	Tennis	38.8 (4.2)	33.4 (13.7)	35.3 (6.7)	42.4 (11.5)	37.5 (4.0)	3/12
F07	Tennis	25.8 (10.7)	14.3 (22.2)	4.3 (5.8)	52.6 (9.5)	24.3 (20.8)	6/12
F08	Basketball	39.9 (44.3)	21.2 (25.0)	13.4 (21.4)	22.2 (32.1)	24.2 (11.2)	9/12
Total female athletes		26.2 (9.5)	26.5 (9.3)	25.2 (16.9)	33.6 (14.0)	27.2 (8.9)	58 (29)%
M01	Cycling	48.1 (10.7)	45.1 (12.1)	25.8 (5.0)	30.8 (5.2)	37.5 (10.8)	4/12
M02	Athletics	37.3 (14.7)	30.3 (24.9)	30.2 (15.2)	40.5 (12.8)	34.6 (5.2)	5/12
M03	Athletics	36.0 (16.6)	19.1 (10.0)	25.8 (2.9)	28.4 (6.4)	27.3 (7.0)	9/12
M04	Cycling	59.1 (15.1)	50.1 (10.2)	48.4 (17.6)	42.8 (19.3)	50.1 (6.7)	2/11
M05	Cycling	50.2 (15.5)	38.3 (7.2)	42.6 (13.0)	54.5 (2.2)	46.4 (7.3)	1/12
M06	Cycling	35.8 (4.6)	30.9 (14.9)	30.6 (6.2)	47.7 (16.5)	36.3 (8.0)	3/12
Total male athletes		44.4 (9.6)	35.6 (11.2)	33.9 (9.4)	40.8 (10.0)	38.7 (8.3)	34 (23)%
All athletes		34.0 (13.1)	30.7 (10.9)	29.2 (14.2)	36.7 (12.5)	32.1 (10.2)	48 (28)%

SD = standard deviation, EA = Energy Availability (kcal/kg fat-free mass/day), and LEA = low energy availability (≤30 kcal/kg fat-free mass/day). T0 = baseline, T1 = 4 weeks, T2 = 8 weeks, and T3 = 12 weeks. “.” = values were missing for F03 at T1 and F04 at T2.

**Table 4:** Blood biochemical parameters.

	T0	T1	T2	T3
Hemoglobin (g/L)	138 (11)	135 (14)	137 (12)	138 (15)
Anemia (n (%))				
Females (hemoglobin < 120 g/dL)	1 (13)	1 (13)	2 (25)	1 (13)
Males (hemoglobin < 140 g/dL)	2 (33)	3 (50)	2 (33)	1 (17)
Iron deficiency (<30 μg/L) (n (%))				
Females	3 (38)	2 (25)	3 (38)	2 (25)
Males	0	0	0	0
Iron deficiency with anemia (n (%))				
Females	1 (13)	1 (13)	2 (25)	1 (13)
Males	0	0	0	0
Insufficient vitamin D (<75 nmol/L) (n (%))				
Females	6 (75)	6 (75)	5 (63)	4 (50)
Males	5 (83)	3 (50)	1 (17)	3 (50)

Data are reported as mean (standard deviations).

## Data Availability

The dataset generated and analyzed during the current study is available from the corresponding author upon reasonable request.
